# Sodium hydrosulphide restores tumour necrosis factor‐α‐induced mitochondrial dysfunction and metabolic dysregulation in HL‐1 cells

**DOI:** 10.1111/jcmm.14637

**Published:** 2019-09-08

**Authors:** Ting‐I Lee, Yu‐Hsun Kao, Lkhagva Baigalmaa, Ting‐Wei Lee, Yen‐Yu Lu, Yao‐Chang Chen, Tze‐Fan Chao, Yi‐Jen Chen

**Affiliations:** ^1^ Department of General Medicine School of Medicine College of Medicine Taipei Medical University Taipei Taiwan; ^2^ Division of Endocrinology and Metabolism Department of Internal Medicine School of Medicine College of Medicine Taipei Medical University Taipei Taiwan; ^3^ Division of Endocrinology and Metabolism Department of Internal Medicine Wan Fang Hospital Taipei Medical University Taipei Taiwan; ^4^ Graduate Institute of Clinical Medicine College of Medicine Taipei Medical University Taipei Taiwan; ^5^ Department of Medical Education and Research Wan Fang Hospital Taipei Medical University Taipei Taiwan; ^6^ Division of Cardiology Department of Internal Medicine Sijhih Cathay General Hospital New Taipei City Taiwan; ^7^ Department of Biomedical Engineering National Defense Medical Center Taipei Taiwan; ^8^ Department of Medicine Heart Rhythm Center and Division of Cardiology Taipei Veterans General Hospital Taipei Taiwan; ^9^ Institute of Clinical Medicine and Cardiovascular Research Center National Yang‐Ming University Taipei Taiwan; ^10^ Division of Cardiovascular Medicine Department of Internal Medicine Wan Fang Hospital Taipei Medical University Taipei Taiwan; ^11^ Cardiovascular Research Center Wan Fang Hospital, Taipei Medical Univsersity Taipei Taiwan

**Keywords:** fatty acid metabolism, HL‐1 cardiomyocytes, peroxisome proliferator‐activated receptors, proinflammatory cytokines, receptor for advanced glycation end, sodium hydrosulphide

## Abstract

Tumour necrosis factor (TNF)‐α induces cardiac metabolic disorder and mitochondrial dysfunction. Hydrogen sulphide (H_2_S) contains anti‐inflammatory and biological effects in cardiomyocytes. This study investigated whether H_2_S modulates TNF‐α‐dysregulated mitochondrial function and metabolism in cardiomyocytes. HL‐1 cells were incubated with TNF‐α (25 ng/mL) with or without sodium hydrosulphide (NaHS, 0.1 mmol/L) for 24 hours. Cardiac peroxisome proliferator‐activated receptor (PPAR) isoforms, pro‐inflammatory cytokines, receptor for advanced glycation end products (RAGE) and fatty acid metabolism were evaluated through Western blotting. The mitochondrial oxygen consumption rate and adenosine triphosphate (ATP) production were investigated using Seahorse XF24 extracellular flux analyzer and bioluminescence assay. Fluorescence intensity using 2′, 7′‐dichlorodihydrofluorescein diacetate was used to evaluate mitochondrial oxidative stress. NaHS attenuated the impaired basal and maximal respiration, ATP production and ATP synthesis and enhanced mitochondrial oxidative stress in TNF‐α‐treated HL‐1 cells. TNF‐α‐treated HL‐1 cells exhibited lower expression of PPAR‐α, PPAR‐δ, phosphorylated 5′ adenosine monophosphate‐activated protein kinase‐α2, phosphorylated acetyl CoA carboxylase, carnitine palmitoyltransferase‐1, PPAR‐γ coactivator 1‐α and diacylglycerol acyltransferase 1 protein, but higher expression of PPAR‐γ, interleukin‐6 and RAGE protein than control or combined NaHS and TNF‐α‐treated HL‐1 cells. NaHS modulates the effects of TNF‐α on mitochondria and the cardiometabolic system, suggesting its therapeutic potential for inflammation‐induced cardiac dysfunction.

## 1 INTRODUCTION

Tumour necrosis factor (TNF)‐α is an adipose‐derived proinflammatory cytokine that critically regulates cardiac function.^1^ TNF‐α induces myocardial contractile dysfunction of the cardiomyocytes during heart failure.^2^ TNF‐α leads to insulin resistance by augmenting lipolysis in adipocytes and thereby increasing the phosphorylation of the serine/threonine of insulin receptor substrate (IRS)‐1.^3^ Nuclear transcription factors including peroxisome proliferator‐activated receptor (PPAR) isoforms α, γ and δ regulate myocardial glucose, lipid and energy homeostasis.^4^ Impaired cardiac energy metabolism is common in myocardial stress because of heart failure, ischaemia or diabetic cardiomyopathy.^5^, ^6^ TNF‐α has been shown to change cardiac expression of PPAR isoforms by increasing oxidative stress.^7^ In addition, diabetes mellitus and hypertension can modulate myocardial PPARs through increased inflammatory cytokines and oxidative stress^8^, ^9^ The receptor for advanced glycation end products (RAGE) is a multiligand receptor that is directly involved in the inflammatory response expressed in several tissues including the heart.^10^, ^11^ Animal studies have demonstrated that RAGE and the ligands of RAGE are overexpressed in heart injuries, diabetes mellitus and inflammation.^12^, ^13^ These findings suggest that inflammation‐induced metabolic disorder may contribute to cardiac dysfunction during stress, such as heart failure, diabetes mellitus or sepsis. Moreover, mitochondria play a vital role in maintaining cardiac function. Previous studies have shown that TNF‐α may impair mitochondrial activity with decreasing adenosine triphosphate (ATP) function and increasing oxidative stress.^14^, ^15^ Sepsis may produce heart failure through dysregulation of cardiac metabolism and mitochondrial function. However, effective treatments targeting adverse cardiac effects of pro‐inflammatory cytokines are limited.

Hydrogen sulphide (H_2_S) is considered the third member of the gasotransmitter family discovered after nitric oxide and carbon monoxide.^16^ Recent studies have shown that H_2_S acts as a crucial mediator in numerous signalling pathways of human biology, such as the blood vessels,^17^ heart,^18^ gastrointestinal tract and central nervous system.^19^ Accumulating evidence has also illustrated the potential benefits of H_2_S in the pathophysiology of the cardiovascular system.^20^, ^21^ H_2_S can attenuate myocardial ischaemia‐reperfusion injury through preservation of mitochondrial function, and this may reduce morbidity and mortality associated with ischaemia‐induced heart failure.^22^, ^23^ Moreover, H_2_S was strongly suggested in several studies to be a potent anti‐inflammatory molecule in various models.^24^, ^25^ The physiological levels of H_2_S in the circulation were reported to be approximately 0.01‐0.1 mmol/L in healthy animals and humans.^26^ Sodium hydrosulphide (NaHS), an H_2_S donor, could generate H_2_S quickly with an HS^−^/H_2_S ratio of around 3:1.^27^ NaHS has been extensively used while studying the biological effect of H_2_S. Therefore, this study investigated whether NaHS modulates TNF‐α‐dysregulated mitochondrial function and metabolism in cardiomyocytes and evaluated the potential underlying mechanisms.

## 2 MATERIALS AND METHODS

### 2.1 Cell preparations

HL‐1 cells were provided by Dr. Claycomb. The cells were derived from mouse atrium with differentiated biochemical and morphological properties of adult atrial cardiomyocytes.^28^ The HL‐1 cells were cultured in a humidified atmosphere of 5% CO_2_ at 37°C in the Claycomb medium (SAFC Biosciences), as described previously.^7^ To perform the experiments, the cells were incubated with and without TNF‐α (25 ng/mL, Sigma Aldrich Inc) for 24 hours, followed by with or without NaHS (0.1 mmol/L, Sigma).^29^

### 2.2 Measurement of cellular ATP levels

Intracellular ATP levels in HL‐1 cells were measured utilizing an ATP bioluminescence assay kit (FL‐AA, Sigma‐Aldrich), as described previously.^30^ Briefly, the ATP in HL‐1 cells is consumed and light is emitted based on the conversion of luciferin to light by firefly luciferase in an ATP‐dependent manner, and the light released is proportional to the ATP present. To measure ATP concentration, all samples were referred to an ATP standard curve with known concentrations ranging from 10^−12^ to 10^−4^ mol/L and then normalized to the number of cells.

### 2.3 Oxidative stress in HL‐1 cardiomyocytes

To detect intracellular reactive oxygen species (ROS) levels, a 2′, 7′‐dichlorodihydrofluorescein diacetate (H2DCF‐DA) ROS Detection Assay Kit (Thermo Fisher Scientific) was used. According to the manufacturer's instructions, HL‐1 cells with or without TNF‐α and NaHS were incubated in dark with 20 μmol/L H2DCF‐DA for 30 minutes at 37°C. Subsequently, the cells were harvested and transferred to a 96‐well microplate. The fluorescence intensity was measured with a fluorescence plate reader (Varioskan Flash reader, Thermo Fisher Scientific) at excitation and emission wavelengths of 492 and 517 nm, respectively. The increase in fluorescence intensity signified up‐regulated cellular ROS levels in the HL‐1 cells.

### 2.4 Mitochondrial oxygen consumption rate

HL‐1 cells with or without TNF‐α and NaHS in an unbuffered Dulbecco's modified Eagle medium at pH 7.4 were transferred at a density of 2 × 10^5^ cells/well to the wells of an XF24 Seahorse assay plate to determine the mitochondrial oxygen consumption rate (OCR) through an XF analysis (XF24, Seahorse Bioscience), as described previously.^30^ Before initiating the assay, the cells were incubated at 37°C without CO_2_ for 1 hour for equilibration. For the measurement of the bioenergetics profile, basal OCR was initially measured in triplicate. Sequentially, the cells were treated with oligomycin (1 μmol/L) to inhibit ATP synthase, and carbonyl cyanide p‐trifluoromethoxy phenylhydrazone (FCCP; 1 μmol/L) was added to yield maximal uncoupled respiration. Non‐mitochondrial respiration was detected by adding rotenone (0.5 μmol/L) and antimycin A (0.5 μmol/L). The optimal concentration of each modulator was measured in concentration response experiments. Under these conditions, we evaluated mitochondrial basal respiration (baseline respiration‐antimycin A+rotenone post‐injection respiration), ATP‐linked respiration (baseline respiration‐oligomycin post‐injection respiration), maximal respiratory capacity (FCCP‐stimulated respiration‐antimycin A+rotenone post‐injection respiration), and reserve respiratory capacity (FCCP‐stimulated respiration‐baseline respiration) of the TNF‐α‐treated HL‐1 cells with or without NaHS.

### 2.5 Western blot analysis

Equal amounts of total proteins from the HL‐1 cells were resolved using sodium dodecyl sulphate‐polyacrylamide gel electrophoresis (SDS‐PAGE), followed by the electrophoretic transfer of proteins onto nitrocellulose membranes. Equal amounts of proteins (40 μg) were resolved using SDS‑PAGE on an 8%‐15% gel, followed by the electrophoretic transfer of proteins onto polyvinylidene difluoride membranes. Blots were blocked with 5% skimmed milk for 1 h at room temperature and then probed with antibodies against PPAR‐γ coactivator 1‐α (PGC‐1α, Abcam, Cambridge, UK), phosphorylated (p) acetyl coenzyme A carboxylase (ACC) (pACC, Millipore), carnitine palmitoyltransferase 1 (CPT‐1, Santa Cruz Biotechnology), diacylglycerol O‐acyltransferase 1 (DGAT1, Abcam), 5′ adenosine monophosphate‑activated protein kinase (AMPK) α2 (EMD Millipore.), phosphorylated (p)‑AMPKα2 Thr172 (EMD Millipore), PPAR‐α (Thermo Fisher Scientific), PPAR‐γ (Santa Cruz Biotechnology), PPAR‐δ (Affinity Bio Reagent), interleukin (IL)‑6 (Thermo Fisher Scientific), RAGE (Thermo Fisher Scientific), glucose transporter (GLUT) 4 (Abcam), IRS‐1 (Cell Signaling Technology), phosphorylated IRS‐1 (pIRS‐1) at Ser307 (Cell Signaling Technology), protein kinase B (Akt, Cell Signaling Technology), phosphorylated Akt (pAkt, Cell Signaling Technology), total and phosphorylated extracellular signal‐regulated kinases (pERK 1/2) (Cell Signaling Technology) and total and phosphorylated α subunit of the inhibitor of _Κ_B (I_Κ_B_α_) and phosphorylated I_Κ_B_α_ (pI_Κ_B_α_) (Santa Cruz Biotechnology) for overnight at 4°C and secondary antibodies conjugated with horseradish peroxidase (Leinco Technologies, Inc.) for 1 hour at room temperature. Bound antibodies were detected using an enhanced chemiluminescence detection system (EMD Millipore) and analysed with AlphaEaseFC software (version 6.0; Protein Simple). Targeted bands were normalized to cardiac glyceraldehyde 3‐phosphate dehydrogenase (Sigma‑Aldrich) to confirm equal protein loading.

### 2.6 Statistical methods

A paired *t* test or Systat software Sigma Pot version 12 (Systat Software Inc.) one‐way analysis of variance (ANOVA) with Duncan's method for multiple comparisons was used to compare differences between groups when appropriate. *P *< .05 was considered to be statistically significant.

## 3 RESULTS

### 3.1 Effect of H_2_S on TNF‐α‐dysregulated ATP synthesis, oxidative stress and mitochondrial function

**Figure 1:**
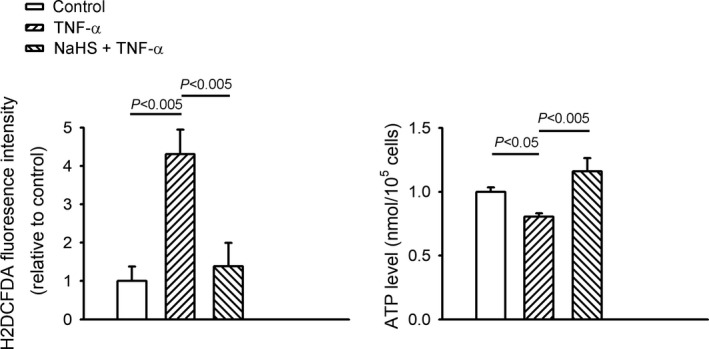
Sodium hydrosulphide (NaHS) decreased oxidative stress and increased adenosine triphosphate (ATP) synthesis in tumour necrosis factor (TNF)‐α‐treated HL‐1 cells. Oxidative stress was measured using a fluorescent dichlorofluorescein assay, and intracellular ATP levels were measured using an ATP bioluminescence assay kit in the control HL‐1 cells and TNF‐α (25 ng/mL)‐treated HL‐1 cells in the presence or absence of NaHS (0.1 mmol/L) for 24 h. Data are shown as mean ± SEM of five independent experiments

As shown in Figure 1, TNF‐α‐treated HL‐1 cells led to lower ATP production than the control HL‐1 cells and the combined NaHS and TNF‐α‐treated HL‐1 cells. The TNF‐α‐treated cells exhibited greater cellular oxidative stress than the controls and HL‐1 cells treated with a combination of NaHS and TNF‐α. Cellular oxidative stress and ATP production were similar in the controls and HL‐1 cells treated with a combination of NaHS and TNF‐α.

**Figure 2:**
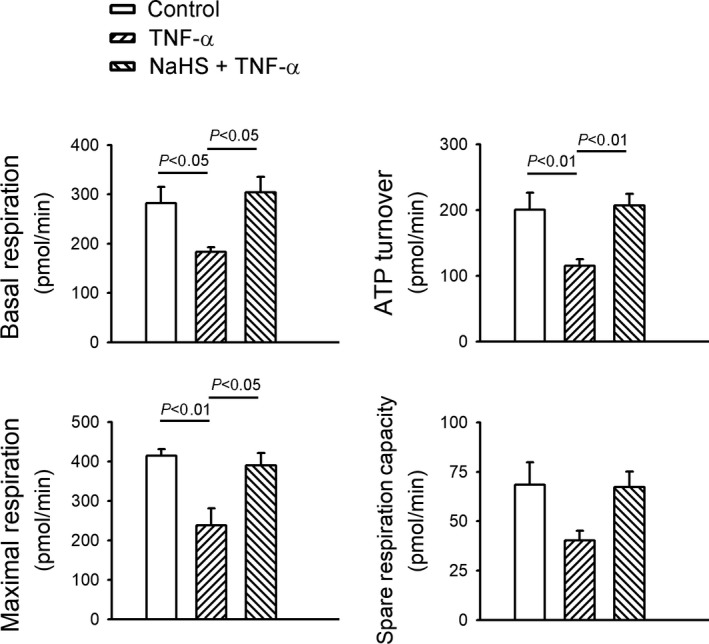
Sodium hydrosulphide (NaHS) improved mitochondrial dysfunction in tumour necrosis factor (TNF)‐α‐treated HL‐1 cells. Oxygen consumption rates and bioenergetics profiles were determined using a XF24 Seahorse analyzer in TNF‐α (25 ng/mL)‐treated cells in the presence or absence of NaHS (0.1 mmol/L) for 24 h. TNF‐α (25 ng/mL)‐treated cells with and without NaHS (0.1 mmol/L). Data of each experiment represent five Seahorse microplate wells

The TNF‐α‐treated HL‐1 cells had significantly lower basal, maximal and ATP‐linked OCR than the control cells and HL‐1 cells treated with a combination of NaHS and TNF‐α (Figure 2). The spare respiratory capacity was similar between the controls, TNF‐α‐treated HL‐1 cells and the HL‐1 cells treated with a combination of NaHS and TNF‐α.

### 3.2 Effect of NaHS on TNF‐α‐mediated myocardial fatty acid and glucose metabolic dysregulation

**Figure 3:**
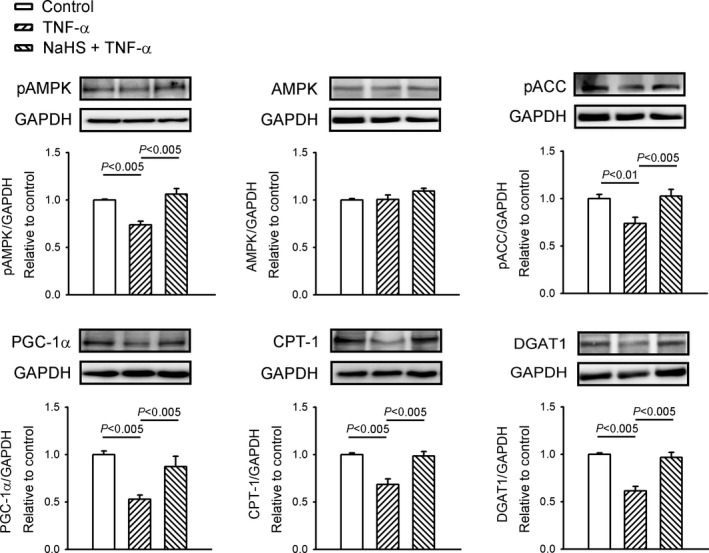
Sodium hydrosulphide (NaHS) improved fatty acid dysregulation in tumour necrosis factor (TNF)‐α‐treated HL‐1 cells. Western blot analysis of 5′ adenosine monophosphate‐activated protein kinase (AMPK) 2α, phosphorylated AMPK2α (pAMPK2α), phosphorylated acetyl coenzyme A carboxylase (pACC), peroxisome proliferator‐activated receptor‐γ coactivator‐1α (PGC‐1α), carnitine palmitoyltransferase 1 (CPT‐1) and diacylglycerol acyltransferase 1 (DGAT1) expression from cells treated with TNF‐α (25 ng/mL) or NaHS (0.1 mmol/L) combined with TNF‐α for 24 h. Densitometry was normalized to glyceraldehyde 3‐phosphate dehydrogenase (GAPDH) as an internal control. Data are shown as mean ± SEM from four independent experiments

As shown in Figure 3, compared with the control HL‐1 cells, the TNF‐α‐treated HL‐1 cells had lower protein expression of pAMPK2α, pACC, PGC‐1α, CPT‐1 and DGAT1, which was ameliorated by co‐administration with NaHS. However, the control HL‐1 cells, TNF‐α‐treated HL‐1 cells and HL‐1 cells treated with a combination of NaHS and TNF‐α had similar protein expressions of total AMPK2α.

**Figure 4:**
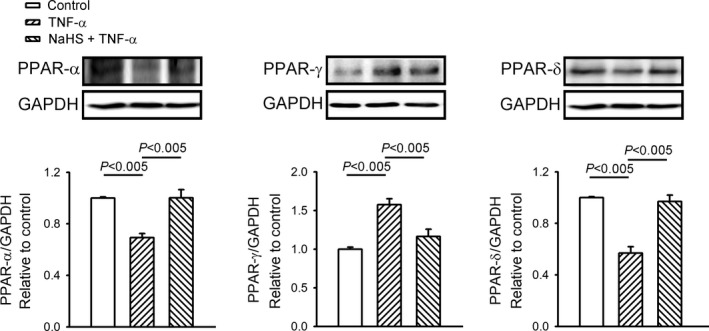
Sodium hydrosulphide (NaHS) reversed the effect of tumour necrosis factor (TNF)‐α on peroxisome proliferator‐activated receptors (PPARs). Representative immunoblots and average data of cardiac PPAR‐α, PAPR‐γ and PPAR‐δ protein levels from cells treated with TNF‐α (25 ng/mL), or NaHS (0.1 mmol/L) combined with TNF‐α for 24 h. Densitometry was normalized to glyceraldehyde 3‐phosphate dehydrogenase (GAPDH) as an internal control. Data are shown as mean ± SEM from four independent experiments

The TNF‐α‐treated HL‐1 cells had lower PPAR‐α protein levels, higher PPAR‐γ expression and lower PPAR‐δ expression than the control HL‐1 cells (Figure 4). The HL‐1 cells treated with a combination of NaHS and TNF‐α and the control HL‐1 cells had similar protein expressions of PPAR‐α, PPAR‐γ and PPAR‐δ.

**Figure 5:**
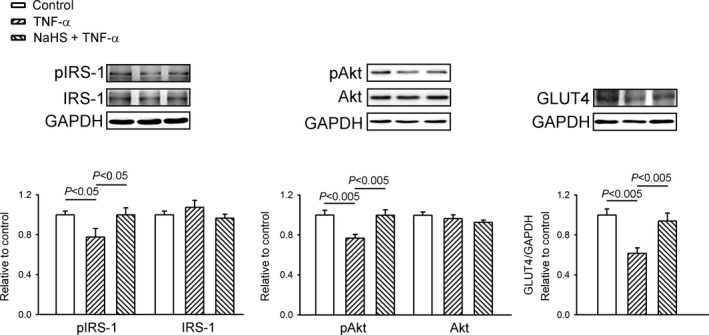
Sodium hydrosulphide (NaHS) modulated the insulin signalling pathway in tumour necrosis factor (TNF)‐α‐treated HL‐1 cells. Representative immunoblots and average data of an insulin receptor substrate (IRS)‐1, phosphorylated IRS‐1 (pIRS‐1), protein kinase B (Akt), phosphorylated AkT (pAkt) and glucose transporter 4 (GLUT4) from cells treated with TNF‐α (25 ng/mL) or NaHS (0.1 mmol/L) combined with TNF‐α for 24 h. Densitometry was normalized to glyceraldehyde 3‐phosphate dehydrogenase (GAPDH) as an internal control. Data are shown as mean ± SEM from four independent experiments

As illustrated in Figure 5, the TNF‐α‐treated HL‐1 cells had lower protein expression of pAkt, pIRS‐1 at Ser307 than control and/or combined NaHS with TNF‐α‐treated HL‐1 cells. However, total Akt and total IRS‐1 were expressed similarly (Figure 5). Additionally, the protein expression of GLUT4 in the TNF‐α‐treated HL‐1 cells was lower than that in the control cells and the HL‐1 cells treated with a combination of NaHS and TNF‐α.

### 3.3 Effects of NaHS on TNF‐α‐mediated myocardial proinflammatory cytokines, RAGE and downstream signalling

**Figure 6:**
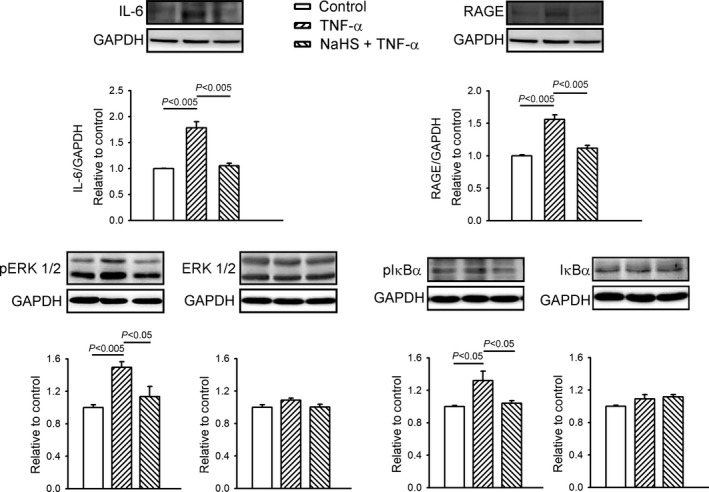
Sodium hydrosulphide (NaHS) attenuated the receptor of advanced glycation end products (RAGE) and inflammatory cytokines in tumour necrosis factor (TNF)‐α‐treated HL‐1 cells. Western blot analysis of RAGE, interleukin‐6 (IL‐6), extracellular signal‐regulated kinases (ERK 1/2), phosphorylated ERK (pERK 1/2), α subunit of the inhibitor of _Κ_B (I_Κ_B_α_) and phosphorylated I_Κ_B_α_ (pI_Κ_B_α_) expression in cells treated with TNF‐α (25 ng/mL) or NaHS (0.1 mmol/L) combined with TNF‐α for 24 h. Densitometry was normalized to glyceraldehyde 3‐phosphate dehydrogenase (GAPDH) as an internal control. Results are from four independent experiments

We studied the effects of H_2_S on TNF‐α‐induced inflammatory signalling. The TNF‐α‐treated HL‐1 cells exhibited greater protein expression of IL‐6 and RAGE than the control HL‐1 cells (Figure 6). However, the protein levels of IL‐6 and RAGE were similar in the controls and HL‐1 cells treated with a combination of NaHS and TNF‐α. Additionally, the TNF‐α‐treated HL‐1 cells exhibited higher protein expression of pERK 1/2 and pI_Κ_B_α_than the control HL‐1 cells. However, the protein expressions of total ERK 1/2 and total I_Κ_B_α_ in the controls and HL‐1 cells treated with a combination of NaHS and TNF‐α were similar.

## 4 DISCUSSION

Several investigations suggested that low‐grade systemic inflammation is closely correlated with metabolic dysregulation.^31^, ^32^ Pro‐inflammatory cytokines increase systematically during low‐grade chronic inflammation^33^ and might be associated with cardiac insulin resistance.^3^, ^34^ Excess‐free fatty acid is considered as one of the major players in the development of chronic inflammation and myocardial damage.^35^ However, few potential targets were available in attenuating the adverse cardiac effects of inflammatory cytokines. In this study, NaHS significantly improved the adverse effects of TNF‐α on mitochondrial function with preserved ATP production, mitochondrial oxygen consumption and oxidative stress. Additionally, NaHS attenuated myocardial lipid dysregulation in the TNF‐α‐treated HL‐1 cells by decreasing the protein expressions of IL‐6, RAGE and its downstream signalling pERK and pIKB protein expressions. These findings suggest the therapeutic potential of H_2_S in inflammation‐induced metabolic dysregulation.

ROS are important contributors to lipid‐induced insulin resistance during inflammation. Mitochondrial ROS production is highly regulated and important for various cell functions. However, high ROS levels are associated with significant cell damage and mitochondrial dysfunction in a process known as oxidative stress,^36^ which is usually associated with the aetiology of obesity, insulin resistance and type 2 diabetes mellitus.^37^ H_2_S plays a critical role in protecting cardiomyocytes against apoptosis,^38^ oxidative stress,^39^ endoplasmic reticulum (ER) stress and autophagy.^40^ In addition, H_2_S is also involved in alleviating diabetic myocardial injury by protecting cardiac mitochondria.^41^ In our study, NaHS improved TNF‐α‐induced mitochondrial dysfunction, which might contribute to its beneficial effects on oxidative stress and ATP synthesis in HL‐1 cardiomyocytes.

Similar to our previous investigations,^42^, ^43^ abnormal myocardial fatty acid metabolic signals were found in the TNF‐α‐treated HL‐1 cells. AMPK plays a crucial role in the myocardial regulation of glucose metabolism.^44^ The decreased cardiac AMPK phosphorylation in TNF‐α‐treated HL‐1 cells may be associated with augmented oversupply of lipid, which can dephosphorylate and deactivate AMPK.^45^ AMPK modulates fatty acid energy metabolism in different ways during myocardial cellular stress.^46^, ^47^ AMPK stimulates the transport of fatty acids into mitochondria through phosphorylation and inhibition of ACC.^48^ We have also discovered that NaHS stimulated AMPK‐2α and ACC phosphorylation. This action countered the inhibition of AMPK‐2α activity and ACC phosphorylation caused by TNF‐α in the cardiomyocytes.

PGC‐1α is required for AMPK to regulate the expression of several key players in mitochondrial and glucose metabolism.^49^ PGC‐1α plays an important role during the regulation of fatty acid β‐oxidation, and the excess accumulation of myocardial lipid might cause worsening of the energy balance between cardiac lipid and glucose utilization.^50^ A suppressed glucose metabolism was found in the inflammation‐related cardiac dysfunction.^51^ Similarly, treatment with TNF‐α decreases PGC‐1α and GLUT4 protein expressions and inhibits the insulin signalling pathway (pAkt, pIRS‐1) in HL‐1 cells. Additionally, the decrease in the CPT‐1 protein level in the TNF‐α‐treated HL‐1cells might have led to an increase in the supply of fatty acids to the mitochondria. The elevation of these metabolic substrates could have resulted in the accumulation of unoxidized fatty acids that can be transformed to intracellular lipid intermediates leading to cardiac lipotoxicity.

PPARs deliver energy in cardiomyocytes by regulating fatty acid β‐oxidation and glucose metabolism. PGC‐1α is coactivated with members of the PPAR nuclear receptor transcription factor superfamily to activate gene expression involved in mitochondrial fatty acid oxidation.^52^ We found a significant decline in both PPAR‐α and PPAR‐δ proteins in parallel to the results of our previous investigations,^9^, ^53^ probably because of the compensatory response to insulin resistance caused by TNF‐α during inflammation.^7^ Similar to our previous studies,^7^, ^53^ our study demonstrated that TNF‐α increases PPAR‐γ expression in cardiomyocytes, which might result in cardiac lipotoxicity through its lipogenic effect. PPAR‐γ was thought in part to regulate DGAT1 to govern lipogenesis and lipid storage.^54^ However, in this study, the TNF‐α‐treated HL‐1 cells had reduced DGAT1 expression compared with the control cells. Although the mechanism underlying this finding is unclear, it is speculated that TNF‐α may reduce DGAT1 expression in cardiomyocytes via PPAR‐γ‐independent signalling. DGAT1 is localized in the ER^55^ and is crucial for preventing cellular lipotoxicity.^56^ Interestingly, in this study, DGAT1 was restored in the cardiomyocytes treated with a combination of TNF‐α and NaHS, suggesting that H_2_S may act as a key regulator of lipotoxicity during myocardial inflammation.

For the first time, we demonstrated that NaHS reverses the inflammatory effects of IL‐6 and RAGE on cardiac PPARs in TNF‐α‐treated HL‐1 cells. As inflammation can regulate expression of PPAR isoforms,^7^, ^9^, ^53^ the effect of NaHS on cardiac PPARs may have been caused by the anti‐inflammatory activity of H_2_S as demonstrated by the decrease in the expression of IL‐6 and RAGE proteins, and its downstream signalling proteins pERK 1/2 and pI_Κ_B_α_. Moreover, NaHS may modulate cardiac metabolism through its effects on PPARs and inflammatory cytokines. However, the experimental setting (HL‐1 cells incubated with NaHS for 24 hours) in this study may not be clinically relevant, because the administration of NaHS to a neutral pH solution leads to an increase in H_2_S levels in minutes, which then reduces to normal levels within 30 minutes to 3 hours.^16^

H_2_S can be produced from cysteine by the pyridoxal‐5′‐phosphate‐dependent enzymes, such as cystathionine β‐synthase and cystathionine γ‐lyase (CSE).^57^ The distribution of H_2_S‐producing enzymes is tissue‐specific, and CSE is a major H_2_S‐generating enzyme in the cardiovascular system.^58^ In mice with ischaemia‐induced heart failure, CSE overexpression in the heart increased H_2_S generation, accompanied by reduced left ventricular dilatation and hypertrophy and improved left ventricular function.^16^ Pharmacological treatment with 1,25 (OH)_2_D_3_ could induce CSE activation and H_2_S formation to increase the total protein amount and membrane translocation of GLUT4 as well as the glucose uptake in high glucose‐treated adipocytes.^59^ A pharmacological approach targeting CSE modulation to increase endogenous H_2_S synthesis may be an effective treatment for cardiovascular diseases. These findings suggest that modulation of endogenous H_2_S production may be a potential therapeutic strategy for cardiometabolic syndrome.

In conclusion, our study elucidated that H_2_S may modulate myocardial PPAR expression through cardiac fatty acid regulation, pro‐inflammatory cytokines, RAGE, oxidative stress and ATP synthesis in TNF‐α‐treated HL‐1 cardiomyocytes.

## CONFLICT OF INTEREST

All authors declare that they have no competing interests.

## AUTHORS CONTRIBUTIONS

TIL was involved in the experimental design, acquisition and analysis of data and drafting of the work. YHK was involved in the conception of the work and critical revision. LB was involved in the experimental design and acquisition of data. TWL was involved in the interpretation of data and critical revision. YYL was involved in the acquisition and interpretation of data. TFC was involved in the analysis and interpretation of data. YCC was involved in the experimental design and interpretation of data. YJC was involved in the conception and experimental design of the work, interpretation of data and critical final revision of the draft. All authors approved the final manuscript and agree to be accountable for all aspects of the work. All persons designated as authors qualify for authorship.

## Data Availability

The data that support the findings of this study are available from the corresponding author upon reasonable request.
